# A Bispecific Antibody Based Assay Shows Potential for Detecting Tuberculosis in Resource Constrained Laboratory Settings

**DOI:** 10.1371/journal.pone.0032340

**Published:** 2012-02-21

**Authors:** Susmita Sarkar, Xinli L. Tang, Dipankar Das, John S. Spencer, Todd L. Lowary, Mavanur R. Suresh

**Affiliations:** 1 Faculty of Pharmacy and Pharmaceutical Sciences, University of Alberta, Edmonton, Alberta, Canada; 2 Departments of Microbiology, Immunology and Pathology, Colorado State University, Fort Collins, Colorado, United States of America; 3 Department of Chemistry and Alberta Ingenuity Centre for Carbohydrate Science, University of Alberta, Edmonton, Alberta, Canada; French National Centre for Scientific Research - Université de Toulouse, France

## Abstract

The re-emergence of tuberculosis (TB) as a global public health threat highlights the necessity of rapid, simple and inexpensive point-of-care detection of the disease. Early diagnosis of TB is vital not only for preventing the spread of the disease but also for timely initiation of treatment. The later in turn will reduce the possible emergence of multi-drug resistant strains of *Mycobacterium tuberculosis*. Lipoarabinomannan (LAM) is an important non-protein antigen of the bacterial cell wall, which is found to be present in different body fluids of infected patients including blood, urine and sputum. We have developed a bispecific monoclonal antibody with predetermined specificities towards the LAM antigen and a reporter molecule horseradish peroxidase (HRPO). The developed antibody was subsequently used to design a simple low cost immunoswab based assay to detect LAM antigen. The limit of detection for spiked synthetic LAM was found to be 5.0 ng/ml (bovine urine), 0.5 ng/ml (rabbit serum) and 0.005 ng/ml (saline) and that for bacterial LAM from *M. tuberculosis* H37Rv was found to be 0.5 ng/ml (rabbit serum). The assay was evaluated with 21 stored clinical serum samples (14 were positive and 7 were negative in terms of anti-LAM titer). In addition, all 14 positive samples were culture positive. The assay showed 100% specificity and 64% sensitivity (95% confidence interval). In addition to good specificity, the end point could be read visually within two hours of sample collection. The reported assay might be used as a rapid tool for detecting TB in resource constrained laboratory settings.

## Introduction

In 2009, the World Health Organisation (WHO) reported 9.4 million cases of tuberculosis (TB) and 1.7 million deaths worldwide [1]. In spite of the best efforts from the researchers round the globe, TB is the leading cause of death by a single, treatable infectious disease. In the last few decades, the situation of TB has worsened due to co-infection with the Human Immune Deficiency (HIV) virus and the emergence of multi-drug resistant (MDR) and extremely drug resistant (XDR) strains of *M. tuberculosis*. In one study, it was estimated that 70% of the AIDS (acquired immunodeficiency syndrome) patients are infected with at least one of the opportunistic mycobacteria pathogens [2]. One of the major reasons for the increased incidence of TB is the lack of a rapid, sensitive, specific, and inexpensive point-of-care (POC) diagnosis for TB. It should be noted that development of new diagnostics for TB has been emphasized as one of the six strategies in the WHO recommendations to combat the spread of the disease [3].

Currently, the diagnosis of TB mainly relies on sputum smear microscopy (SSM), which often gives false negative results. In addition, clinical symptoms and results of chest x-rays are non-specific, making them less reliable. In recent times, more advanced molecular assays like the interferon gamma release assay (IGRA) and the nucleic acid amplification (NAA) assay have been developed but their utility as a POC assay is questionable [4]–[6]. It should be noted, however, that a recent study concluded that in resource-limited environments, a combination of sputum smear microscopy and Xpert-MTB/RIF (an NAA assay) led to both higher accuracy and lower cost of the diagnosis, compared to when the use of a single one of these assays [7]. Nevertheless, these assays can be difficult to perform in resource-constrained countries due to their high cost and technical sophistication. The culture method still remains the gold standard for detection of TB. However, it takes 2–6 weeks to obtain the results, which limits the ability of the health care system to contain the infection and provide suitable medical intervention to the patient in a timely manner. A good number of reviews are available in literature that discusses the advantages and disadvantages of various available detection tests for TB and criteria for an emerging detection assay [8]–[11]. The impact of POC assay is even greater in resource-constrained settings especially as 86% of the TB cases are reported from Asia and sub-Saharan Africa [1]. Furthermore, a POC diagnosis of TB could facilitate proper evaluation of new anti-tuberculosis vaccine trials [12].

Early diagnosis of TB is of paramount importance because TB is highly contagious during the active stage of the disease. A rapid and specific detection of the disease is therefore vital to contain the spread of TB and at the same time will help physicians to initiate proper and timely treatment, which in turn can reduce the chance of bacterial evolution towards MDR and XDR strains [13]. Recent research has led to the identification of several new antigens that could be used for the diagnosis of TB. One such antigen is lipoarabinomannan (LAM), an important non-protein antigen that constitutes 40% of the mycobacterial cell wall [14], and which can modulate the immune response of the host [15]–[18]. Several studies have shown the presence of LAM in different body fluids like sputum, blood and urine during TB [19]–[21]. In addition, LAM is also present in pleural fluid and cerebrospinal fluid of patients with extra pulmonary TB [22]–[24]. These findings highlight the importance of this antigen as a diagnostic marker. Detection of antigen almost certainly confirms the presence of active disease. In contrast, detection of antibody does not always indicate active disease [25] as the antibody response can be detected even six months after clearance of the infection.

The objective of this study was to develop an enzyme-linked immunosorbent assay (ELISA)-based sensitive and specific immunoassay for TB detection. The ELISA was transferred onto an immunoswab platform due to its advantage of visual end point detection, which eliminates the need of using any instrument. The design of the immunoswab assay is based on detection of the LAM antigen. A bispecific monoclonal antibody (bsMAb) capable of simultaneously binding LAM and the enzyme, horseradish peroxidase (HRPO), was developed and used. One of the major advantages of using bsMAb is that after two stage purification, it is already tagged with the reporter enzyme so an extra step of enzyme addition and the subsequent washing steps can be avoided in the assay involving bsMAb [26]. This makes the assay faster compared those using only monoclonal antibodies (MAb). The immunoassay was also evaluated in another format using a chemically conjugated MAb to compare the sensitivity of the assay.

The assay was initially designed with a synthetic epitope of LAM [27] spiked in rabbit serum and bovine urine and later evaluated with native LAM. The assay was also evaluated with stored clinical serum samples collected from TB patients. The use of bsMAb, the first of its kind to detect any TB antigen, in the immunoswab assay provided enhanced sensitivity with a clear background.

## Materials and Methods

### 2.1 Materials

Cell line: YP4, the anti-HRPO rat hybridoma cell line, and the hybridoma cell line that produces MAb CS-35, a murine anti-LAM antibody, were used in this study. YP4 was a kind gift from the late Dr. C. Milstein, Medical Research Council for Molecular Biology, Cambridge, United Kingdom while the cell line producing CS-35 was obtained from Dr. J. S. Spencer, Departments of Microbiology, Immunology and Pathology, Colorado State University, USA. CS-35 is a well-characterised MAb that specifically binds to a motif at the nonreducing terminus of mycobacterial LAM [28]–[29].

Other materials and reagents: Cell culture media RPMI 1640 and Penicillin-streptomycin-glutamine (PSG) were purchased from Gibco (New York, USA). Fetal bovine serum (FBS) was purchased from PAA laboratories (Pasching, Austria). Goat anti-mouse IgG conjugated to horseradish peroxidase (GAM-HRPO), bovine serum albumin (BSA), polyethylene glycol (PEG) 1300–1600, fluorescein isothiocyanate (FITC), tetramethylrhodamine isothiocyanate (TRITC), HRPO Type IV, Protein G-agarose, *m*-amino phenyl boronic acid (*m*-APBA) agarose, and long chain sulfosuccinimidyl NHS biotin were purchased from Sigma Chemicals (St. Louis, USA). Streptavidin tagged HRPO (St-HRPO) was purchased from BD Biosciences (California, USA). Tetramethylbenzidine (TMB) was purchased from BioFx Laboratory (North Carolina, USA). For Western blots, hybond-ECL nitrocellulose membranes were procured from Amersham Biosciences, Germany and the blot detection system was procured from GE Healthcare (USA). Nylon fibre swabs were bought from Micro Rheologics (Brescia, Italy). Non-sterile flat bottom NUNC maxisorp 96-well ELISA plates were purchased from VWR (Ontario, Canada). Fluorescence activated cell sorter, FACSAria (BD Biosciences, USA), was accessed from the Department of Medical, Microbiology and Immunology, University of Alberta. For protein purification, we used a Biologic Duoflow system (Bio-Rad, USA) and the ELISA absorbance was taken using Versa max microplate reader (Molecular Devices, USA).

Rabbit serum was obtained from the Health Sciences Laboratory Animal Services (HSLAS), University of Alberta. Bovine urine was kindly arranged by Dr. Hoon H. Sunwoo, Faculty of Pharmacy and Pharmaceutical Sciences, University of Alberta.

Antigens: For the majority of this study (except the work described in section 2.10), a chemically-synthesized hexaarabinofuranoside epitope [27] of the LAM antigen conjugated to BSA (BSA-Hex) was used. The loading of the hexaarabinofuranoside on BSA was determined by MALDI-MS to be 15.2 saccharides/BSA. Although CS-35 MAb was generated against LAM from *M. leprae* [30], previous studies have demonstrated that this hexasaccharide epitope of the LAM antigen binds strongly to it [31]–[32]. The BSA-Hex was used only used to design the assay. Native LAM from *M. tuberculosis* H37Rv strain (used in section 2.10) was obtained from the Biodefense and Emerging Infections Research Resources Repository Manassas, Virginia, USA listed at http://www.beiresources.org/TBVTRMResearchMaterials/tabid/1431/Default.aspx.

Clinical samples: A total of 21 human serum samples (7 negative and 14 positive) were evaluated. The positive samples were collected from non-HIV cavitary TB patients at diagnosis prior to initiating drug therapy who were part of a cohort of newly diagnosed TB patients from the Tuberculosis Trials Consortium (TBTC) Study Group 22 between 1995 and 1998 and all 14 samples were confirmed positive by culture of a respiratory specimen [33]. The negative samples were collected from healthy U.S. born, non-BCG vaccinated individuals from the Fort Collins, CO, U.S.A. area in 2002. Serum samples from all sources were anonymized and coded to protect donor identities and were obtained with informed consent and/or with permission from the institutional review boards of the relevant institutions involved. The anti-LAM titer for all of these serum samples were characterized by ELISA and Western blot in a recent study [34].

### 2.2 Purification and biotin labelling of CS-35 producing MAb

The expansion, purification and characterisation of the CS-35 MAb were performed according to the published protocol [35]–[36]. The biotin labelling of affinity purified CS-35 was also done as per published protocol [35].

**Figure 1:**
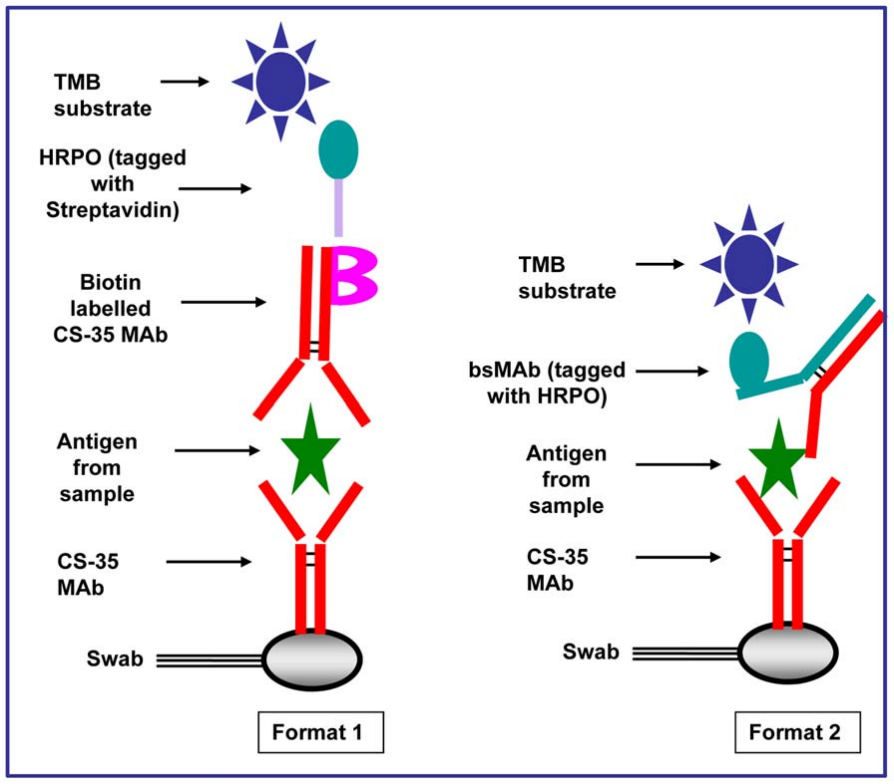
Two formats of sandwich ELISA. **Format 1:** Monoclonal antibody (MAb) based, where both the capture and detection antibody are MAb's. **Format 2:** Bispecific Monoclonal antibody (bsMAb) based, where the capture antibody is a MAb but the detection antibody is a bsMAb.

Purified CS-35 protein was subsequently used in assays (in both formats; refer to Fig. 1) as the capture antibody. The biotin labelled protein was employed as detection antibody in the MAb based immunoswab assay (format 1, Fig. 1).

### 2.3 Development of quadroma cell line

The quadroma cell line was developed by fusing two hybridoma cell lines, the one producing CS-35 and the other YP4. The fusion procedure was performed following published protocols [37]–[39] with slight modifications. Briefly, 2×10^7^ cells were isolated separately from the two cell lines in their logarithmic growth phase; CS-35 (re-suspended in RPMI media, pH 7.4) was labelled with the red dye TRITC and YP4 (re-suspended in RPMI media, pH 6.8) was labelled with the green dye FITC, and incubated for 30 minutes at 37°C and 5% CO_2_. The excess dye was removed by performing repeated washes with RPMI serum free media. The cells were then mixed well, centrifuged at 459× *g* for 7 minutes and the cell pellet was collected. The fusion of the two cell lines was performed by drop-wise addition of 2 ml of PEG to the cell pellet with continuous stirring for 2 minutes at 37°C. The toxic effect of PEG was immediately minimised by diluting the mixture with 20 ml of serum free RPMI media. This mixture was then centrifuged at 114× *g* for 5 minutes and the cell pellet was suspended in RPMI media supplemented with 10% FBS. The fused cells were sorted by florescence-activated cell sorting (FACS) and the dual positive cells were seeded in a 96-well sterile tissue culture plate at a concentration of 1 cell/well. The cells were cultured in 20% FBS media at 37°C with 5% CO_2_ and their growth was routinely monitored under the microscope. Depending on cell growth, after approximately 9–12 days of culture, the cells were screened for their specific activity using bridge ELISA technique.

### 2.4 Bridge ELISA and sub cloning of quadroma cell line

The fused quadroma cells generally secrete three stable antibodies, the two parent MAbs (CS-35 and YP4) and the newly fused bsMAb [26], [40]. To screen for clones that predominantly secrete bsMAb, a bridge ELISA technique was employed [41]. The clones with maximum bsMAb secretion capacity were identified and recloned by the standard limiting dilution method. Briefly, the cells were plated in a tissue culture plate at a concentration of 1 cell/well. They were then cultured as before and positive clones were screened using bridge ELISA. The above cloning and screening steps were repeated until a stable clone (when cloning efficiency >80%, for details see the results) was obtained.

### 2.5 Purification of bsMAb

The bsMAb producing cells were seeded in a hyper flask for large-scale expansion, after 7–10 days the supernatant was harvested and centrifuged at 5000 rpm for 30 minutes. The collected supernatant was passed through a 0.22 µm filter to remove cell debris and the clarified supernatant was further processed to obtain pure bsMAb. A previously published two-stage purification protocol, developed in our lab, was followed [26]. The thus purified bsMAb was used as detection antibody in the bsMAb based immunoswab assay (format 2, Fig. 1).

### 2.6 Optimization of the assay parameters:

The different parameters of the assay such as concentrations of capture antibody (CAb), detection antibody (DAb) and St-HRPO, were optimized separately. Optimization of the assay parameters for format 1 (Fig. 1) is discussed below; assay parameters for format 2 were optimized in a similar manner.

#### 2.6.1 Optimization of the CAb concentration

Purified CS-35 MAb was employed as CAb in the assay. A microtitre plate was coated overnight at 4°C with 100 µl of different concentrations of CS-35 MAb ranging from 0 to 16 µg/ml (0, 1, 2, 4…16) in triplicate. The unbound binding sites on the plate were blocked with 250 µl of 3% dialysed BSA (DBSA) in PBS at 37°C for 3 hours. 100 µl of 5 ng/ml BSA-Hex antigen was added and incubated for 2 hours, and subsequently 4 µg/ml of biotin labelled antibody (DAb) was added and incubated for 1 hour. To this St-HRPO (1∶ 5000 dilution) was added and the plate was incubated for 30 minutes. The plate was washed 3–5 times with PBST after each of steps described above. Finally, TMB was added and the developed colour was read at 650 nm using the microplate reader. The mean of three readings for each concentration was plotted against the corresponding concentration using Microsoft Excel.

#### 2.6.2 Optimization of the DAb concentration

Biotin labelled CS-35 MAb was used as the detection antibody. A fixed concentration of CAb (8 µg/ml) was used to coat a microtitre plate and varied concentrations of DAb ranging from 0 to 16 µg/ml (0, 1, 2, 4…16) were used. The assay protocol and the concentration of the other parameters were same as described in section 2.6.1 and the data were similarly analyzed.

#### 2.6.3 Optimization of the conjugate St-HRPO dilution

Serial two-fold dilutions of the conjugate St-HRPO (in PBS with 1% BSA) ranging from 1∶4,000 to 1∶48,000 were used in the assay. The previously optimised concentrations of the other components such as CAb (8 µg/ml), DAb (2 µg/ml) and BSA-Hex antigen (5 ng/ml) were kept constant. The assay was performed as described in section 2.6.1 and the data was similarly analyzed.

### 2.7 Optimization of BSA-Hex antigen detection sensitivity

The sensitivity of the assay was determined by measuring the lowest amount of the antigen that can be detected by the assay. Different concentrations of the BSA-Hex antigen ranging from 15 ng/ml to 0 (15, 7.5……0) were used together with above optimised concentration for CAb, DAb and conjugate St-HRPO. The assay was performed in triplicate and repeated twice. All the assays reported up until now was performed in microtitre plate. In the subsequent sections we reported the assays using the immunoswab.

### 2.8 Immunoswab assay

Both the MAb- and the bsMAb-based assays (format 1 and 2, respectively, Figure 1) were carried out in the immunoswab format. The swabs were coated with purified CS-35 (50 µL of 25 µg/ml in PBS) at RT for 30 min, dried for 5 minutes and fixed with 50 µL of 95% ethanol. The swabs were then blocked with 5% DBSA in PBS at RT for 45 minutes and were washed five times using PBST with one minute incubation in each washing step. Washes were performed by a simple fill and aspiration method in a microfuge tube. The washed swabs were incubated for 30 min with different concentrations of BSA-Hex antigen in PBS containing 1% DBSA. Subsequently the swabs were washed and incubated for 30 minutes with either bsMAb (50 µL of 25 µg/ml in PBS containing 1% DBSA) in the bsMAb-based assay or with biotin labelled CS-35 (50 µL of 4 µg/ml PBS containing 1% DBSA) in the MAb-based assay. In the bsMAb-based assay, swabs were washed five times as mentioned above and finally 100 µL of TMB was added for colour development. On the other hand, in the MAb-based assay, the swabs were incubated with St-HRPO (1∶10000 dilution in PBS containing 1% DBSA) for 30 min and were washed before TMB was added. The endpoint in both formats was detected visually and compared with the blank swab that was treated as described above except that it was incubated in PBS without any antigen. Following colour development the swabs were scanned using an Epson scanner.

### 2.9 Spiking of antigen in different matrices

Three different solute matrices, namely normal saline, rabbit serum and bovine urine were used to spike the BSA-Hex antigen and then the immunoswab assay was performed to check the limit of detection in each matrix. The assay was performed as per above protocol (section 2.8) in the bsMAb format. The concentration range for the antigen was chosen in the 0–5 ng/ml range. Two swabs were tested in each concentration.

### 2.10 Determination of the limit of detection of whole LAM

The sensitivity of the assay (in bsMAb format) to detect whole LAM isolated from a virulent strain of *M. tuberculosis*, H37Rv, was determined. Different concentrations of the whole LAM antigen were spiked in rabbit serum and the immunoswab assay was performed in duplicate to test the limit of detection.

### 2.11 Determination of assay specificity

#### 2.11.1 Assay performed with related synthetic carbohydrate antigens

The specificity of the immunoswab assay was determined using two different sugars, sucrose and BSA-conjugated to the human blood group A trisaccharide (a kind gift from Professor David R. Bundle, Department of Chemistry, University of Alberta, Canada), together with the TB specific antigen (BSA-Hex). Previous studies have established that the human blood group A trisaccharide does not bind to CS-35 [28]. The immunoswab assay was performed as described above. All three antigens were spiked in saline at 5 µg/ml concentration and the assay was performed in duplicate using a higher concentration range to check for cross reactivity.

#### 2.11.2 Assay performed with different bacterial and fungal culture

The assay was also separately evaluated using the *E.coli* and yeast whole cell culture. The protocol was same as before but here the swabs were incubated in the bacterial and fungal culture after coating and blocking.

#### 2.11.3 Assay performed with different native bacterial and fungal carbohydrate antigens

The cross reactivity of the assay was also checked by using different native carbohydrate antigens. To check for cross-reactive responses to related mycobacterial LAMs, we used LAM from *Mycobacterium smegmatis* and *Mycobacterium leprae* (both were obtained from the Biodefense and Emerging Infections Research Resources Repository Manassas, Virginia, USA). As a related fungal antigen, we used crude cell wall extract from *Candida albicans* (a kind gift from Professor David R. Bundle, Department of Chemistry, University of Alberta, Canada). Due to limited availability of these native antigens, we performed a dot blot assay to check the cross reactivity of the bsMAb. Briefly, two dots were made on nitrocellulose membrane for each of the antigens. The amount of antigen per dot was 1 µg. The dots were allowed to blot on the membrane for 5 min. Then the membrane was blocked with 5% skim milk for 45 min. Next it was washed with PBST for 3 times and 3 ml of bsMAb (4 µg/ml) was added and incubated for 45 min. The membrane was washed 5 times with PBST buffer and the color was developed using TMB substrate. As positive control, we used BSA-Hex.

### 2.12 Evaluation of the assay with the clinical samples

A simple processing procedure was developed to reduce any antibody–antigen immune complexes present in the sera (see discussion below). The optimization was performed in the simulated samples and the optimized condition was tested with the actual samples.

#### 2.12.1 Optimization of the processing conditions

CS-35 was spiked in the rabbit serum (RS) along with the native LAM. This was done to simulate the clinical samples. The MAb was spiked at three different concentrations – 20 µg/ml, 2 µg/ml and 0.2 µg/ml and LAM was used at a concentration of 0.5 ng/ml in all cases. Thus simulated samples were kept at 4°C overnight to facilitate binding between the LAM antigen (Ag) and antibody (Ab). The samples were then treated with different conditions as described in Table 1. After processing, the samples were tested for the antigen level by the developed immunoassay.

**Table 1:** Different processing conditions for simulated serum samples.

Conditions	Reagents used	Temperature & time
1. No treatment	Water = 50 µl	RT for 10 min
2. Glycine+Tris	Glycine (0.1 M, pH 2.3) = 40 µlTris (1 M, pH 9.0) = 10 µl	RT for 10 min, First glycine was added and incubated for 5 min before the solution was neutralised with Tris
3. Urea+heat	Urea (8 M) = 50 µl	93°C for 5 min followed by immediate cooling to 4°C.
4. SDS+heat	SDS (10%) = 20 µlWater = 30 µl	93°C for 5 min followed by immediate cooling to 4°C.
5. SDS+*β*-ME+heat	SDS (10%) = 20 µlWater = 28 µl*β*-ME = 2 µl	93°C for 5 min followed by immediate cooling to 4°C.

The abbreviations used, RT = Room temp, SDS = Sodium dodecyl sulphate, *β*-ME = *β*-mercaptoethanol.

#### 2.12.2 Processing of the clinical samples

In the evaluation of the clinical samples, each was diluted 1∶8 and was then treated with urea and heat as specified in Table 1. Each sample was tested in duplicate.

## Results

### 3.1. Purification and biotin labelling of CS-35 producing MAb

**Figure 2:**
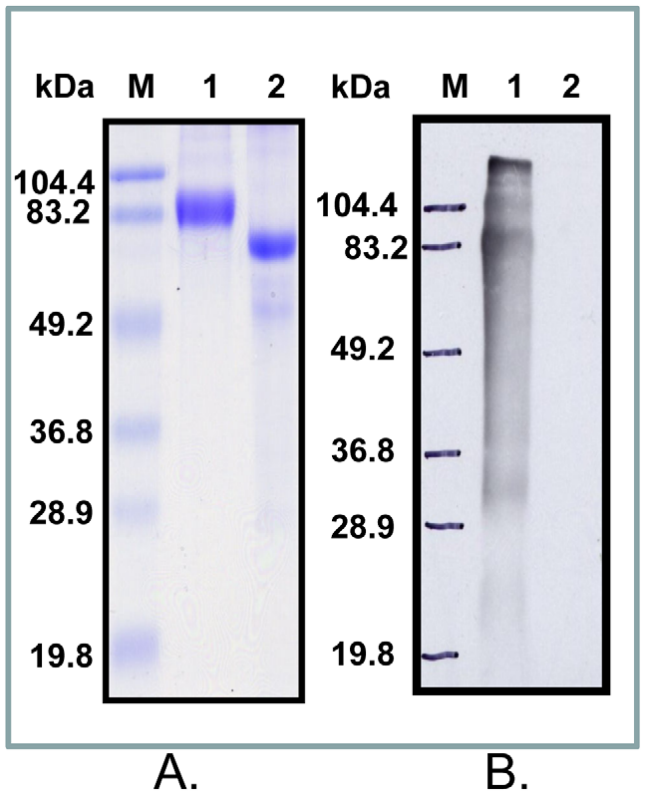
SDS-PAGE and Western blot of BSA-Hex and unconjugated BSA. **A:** Coomassie stained SDS-PAGE gel showing BSA-Hex, which contains 15.2 sugar residues per carrier, and unconjugated BSA, running at 81.4 kDa and 66.3 kDa, respectively. Lane M, standard molecular marker; lane 1, BSA-Hex; lane 2, BSA. **B:** Probe of BSA-Hex and BSA after transfer to nitrocellulose with MAb CS-35 in a Western blot showing reactivity only with BSA-Hex.

Purified CS-35 protein was analysed for binding to the BSA-Hex by Western blotting. BSA-Hex (81.4 kDa) and BSA (66.3 kDa) were run on SDS-PAGE (Fig. 2A.) and transferred to nitrocellulose membrane, which was then probed with purified CS-35 MAb (2 µg/ml). The goat anti-mouse IgG HRPO was used as secondary antibody. A distinct band was observed only towards BSA-Hex with no reactivity towards unconjugated BSA, confirming the specificity of CS-35 MAb towards the hexasaccharide moiety of the LAM antigen (Fig. 2B).

Purified CS-35 MAb was labelled with biotin and labelling was confirmed by a dot blot assay that was performed on a nitrocellulose membrane. Both labelled and unlabelled MAb were placed as dots on the membrane, unbound binding sites were blocked and St-HRPO was added followed by TMB. A blue colour, which is representative of a positive result, developed only in the biotin labelled MAb dots confirming CS-35 MAb biotinylation (data not shown).

### 3.2 Quadroma development

The dual labelled fused cells were sorted by FACS and the fusion efficiency was found to be 0.9%. The positive cells were screened by bridge ELISA and were repeatedly cloned by the limiting dilution method. Cloning efficiency (n) was calculated at each stage by using the following formula:
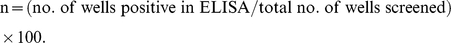



A gradual increase in both activity and cloning efficiency was observed with each subsequent cloning stage. At the end of the process two different quadroma clones (having cloning efficiency >87%) secreting bsMAb were isolated.

### 3.3 Purification of bsMAb

As mentioned before that the two-stage purification of bsMAb was performed by as per published protocol [26]. The purified fraction from the first step contained a mixture of three antibodies, two parent MAbs (CS-35 and YP4) and bsMAb, all of them being the IgG isotype. A second step of purification was performed using *m*-amino phenyl boronic acid (*m*-APBA) agarose to get remove the CS-35 MAb because its presence could affect the sensitivity of the assay. The bsMAb, obtained from *m*-APBA column, was attached to the reporter molecule HRPO via the HRPO-binding paratope. After two stages of purification an increased bsMAb activity was observed in the ELISA activity assay. The extent of purification was confirmed by SDS-PAGE; three distinct bands were observed: heavy chain (∼50 kDa) and light chain (∼25 kDa) of IgG antibody, and HRPO (∼44 kDa) (data not shown).

### 3.4 Optimization of assay parameters

**Figure 3:**
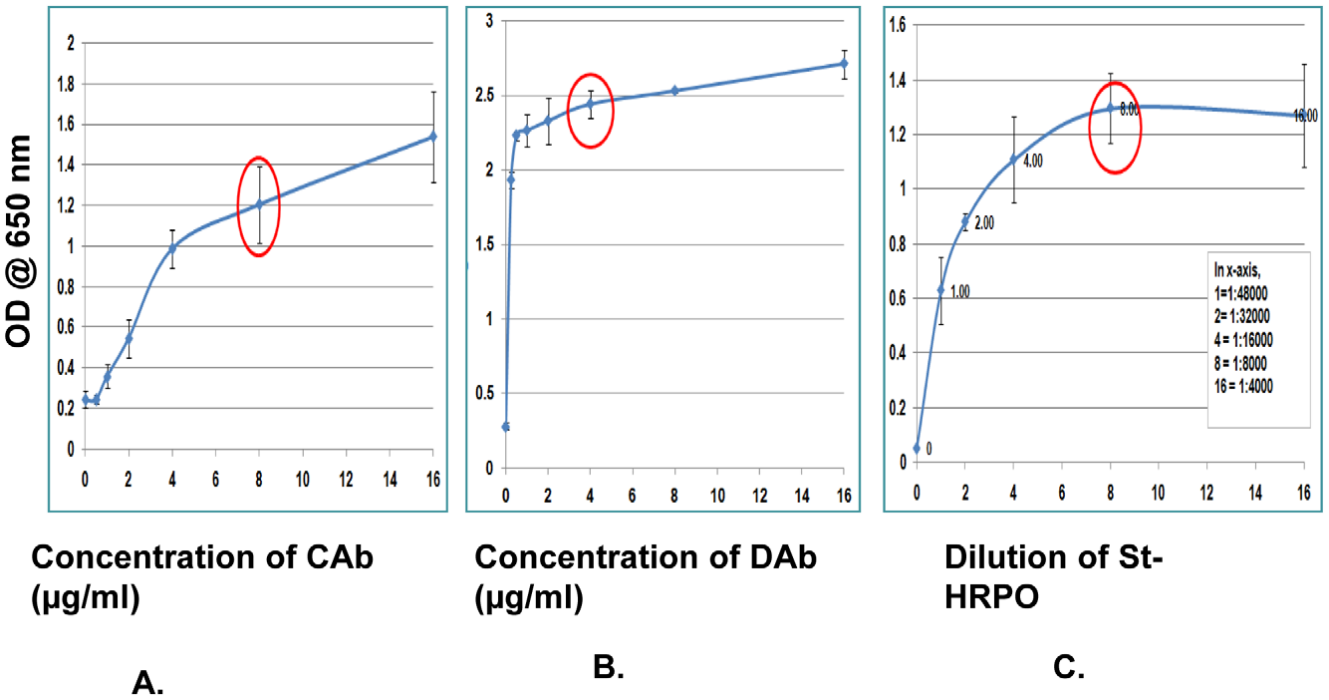
Optimization of different assay parameters. **A:** Optimisation of the capture antibody (CAb), where the concentration 8 µg/ml was found to be optimum. **B:** The optimisation of detection antibody (DAb), where the concentration 4 µg/ml was found to be optimum. **C:** The optimisation of conjugate (st-HRPO), where the dilution 1∶8,000 was found to be optimum.

After obtaining the purified CS-35 MAb, biotinylated CS-35 MAb, and bsMAb, different parameters of the assay were optimized by sandwich ELISA (Fig. 1). Three different sets of assays with different concentrations of capture antibody (CAb), detection antobody (DAb) and St-HRPO were performed in triplicate and repeated twice. In each set, only one parameter was changed while the others were kept constant. The average of three absorbance values was plotted against the respective concentration using Microsoft Excel. In general, for each parameter of the assay, there was an initial steady increase in absorbance value with increasing concentration followed by a slow increase which ultimately merged towards saturation becoming almost parallel to the concentration axis. The point of transition between steady increase and slow increase was chosen as the optimal condition for the respective parameter. Only one set of the results for the optimization of assay parameters was shown in Figure 3. The optimal concentration was found to be 8.0 µg/ml for CAb (Fig. 3A.), 4.0 µg/ml for DAb (Fig. 3B.) and 1∶8000 dilution for St-HRPO (Fig. 3C.).

### 3.5 Antigen detection limit

The assay was evaluated to determine the lowest amount of BSA-Hex antigen that could be detected by the assay. The assay was performed using optimized conditions with different concentrations of BSA-Hex starting from 15 ng/ml. After calculating the statistical significance using Student t-test, 234 pg/ml was found to be the limit of detection in a microtitre plate where p<0.05.

### 3.6 Comparison between biotinylated CS-35 and bsMAb-based immunoswab format

The immunoassays were evaluated in both monoclonal- and bispecific-based formats (Figure 1). The MAb-based assay (biotin–CS-35) showed reduced sensitivity (5 ng/ml) than bsMAb format (0.005 ng/ml) (data not shown). The high background with the MAb-based approach can be explained by the following facts. First, the chemical conjugation of the MAb is a random process where the combination ratio between the MAb and the conjugating chemical (biotin) is varied. In contrast, the bsMAb has two different paratopes, one of which recognises the antigen of interest, LAM and the other paratope binds to a reporter molecule such as HRPO. Therefore, the ratio between the bsMAb and the enzyme is always 1∶1, which helps to provide a clear background. Second, due to the random nature of this chemical conjugation, the site of conjugation is also not fixed and as a result it may block the antigen-binding site on the MAb, which leads to reduced sensitivity. The higher sensitivity of bsMAb format prompted us to use this format for the rest of the study.

### 3.7 Antigen detection limit in different matrices using swab

**Figure 4:**
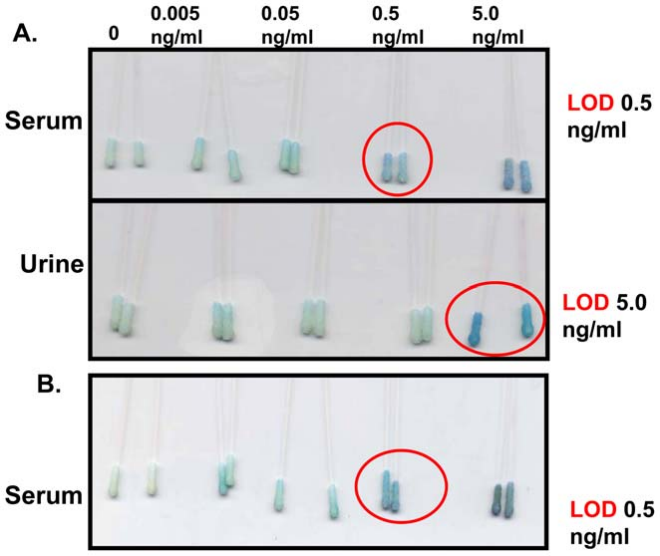
Immunoswab in different matrices. **A:** The immunoswab performed after spiking the BSA-Hex antigen in serum and urine and the limit of detection (LOD) was found to be 0.5 and 5.0 ng/ml in respective matrices (encircled in red). **B:** The immunoswab performed after spiking the bacterial LAM isolated from *M. tuberculosis* H37Rv, in serum and the LOD was found to be 0.5 ng/ml.

Three different solution matrices, normal saline, rabbit serum and bovine urine, were chosen to determine the limit of detection of the antigen in each. This was done because during TB disease the LAM antigen is present in blood and urine. Two swabs were tested in each matrices. The limit of detection of BSA-Hex spiked in saline was found to be 0.005 ng/ml in bsMAb-based assay, while it was lower in serum and urine, 0.5 ng/ml and 5 ng/ml, respectively (Fig. 4A).

### 3.8 Sensitivity of the assay using whole LAM

After the preliminary design of the assay using the chemically synthesised antigen, the immunoswab assay was evaluated for its sensitivity towards whole LAM antigen isolated from a virulent strain of *M. tuberculosis* H37Rv strain. The whole LAM antigen was spiked in rabbit serum and the immunoswab assay was performed in the bsMAb format. The limit of detection of the assay was found to be 0.5 ng/ml (Fig. 4B.). The native LAM was tested only in serum as the designed assay was found to be more sensitive in serum than in urine.

### 3.9 Specificity of the assay

The complete specificity analysis was summarised in Table 2. The designed assay showed no cross reactivity with the structurally unrelated synthetic antigens (sucrose and BSA-conjugated to the human blood group A trisaccharide). In addition, no colour was detected in the assay performed with the *E.coli* and yeast culture. Similar results were obtained in the dot blot assay, against the native candida antigen. However, the antibody showed reactivity with native LAM from both *Mycobacterium smegmatis* and *Mycobacterium leprae*, which would be expected because CS-35 recognizes an epitope common to all LAMs.

**Table 2:** Specificity study of the assay.

Category	Antigen	Results	
		Immunoswab	Dot blot
Synthetic carbohydrate	Sucrose	−ve	NP
	BSA-conjugated to the human blood group A trisaccharide	−ve	NP
	BSA-Hex (specific antigen)	**+ve**	**+ve**
Bacterial culture	*E.coli* (strain HB2151)	−ve	NP
Fungal culture	Yeast (strain A364A)	−ve	NP
Native carbohydrate antigens	LAM from *Mycobacterium smegmatis*	NP	**+ve**
	LAM from *Mycobacterium leprae.*	NP	**+ve**
	Crude cell wall extract from *Candida albicans*	NP	−ve

The specificity was evaluated either of the two formats – immunoswab or dot blot. +ve = blue colour developed; −ve = no blue colour. NP means not performed in the corresponding format. The +ve outcomes are in bold.

### 3.10 Evaluation of the assay with clinical samples

**Figure 5:**
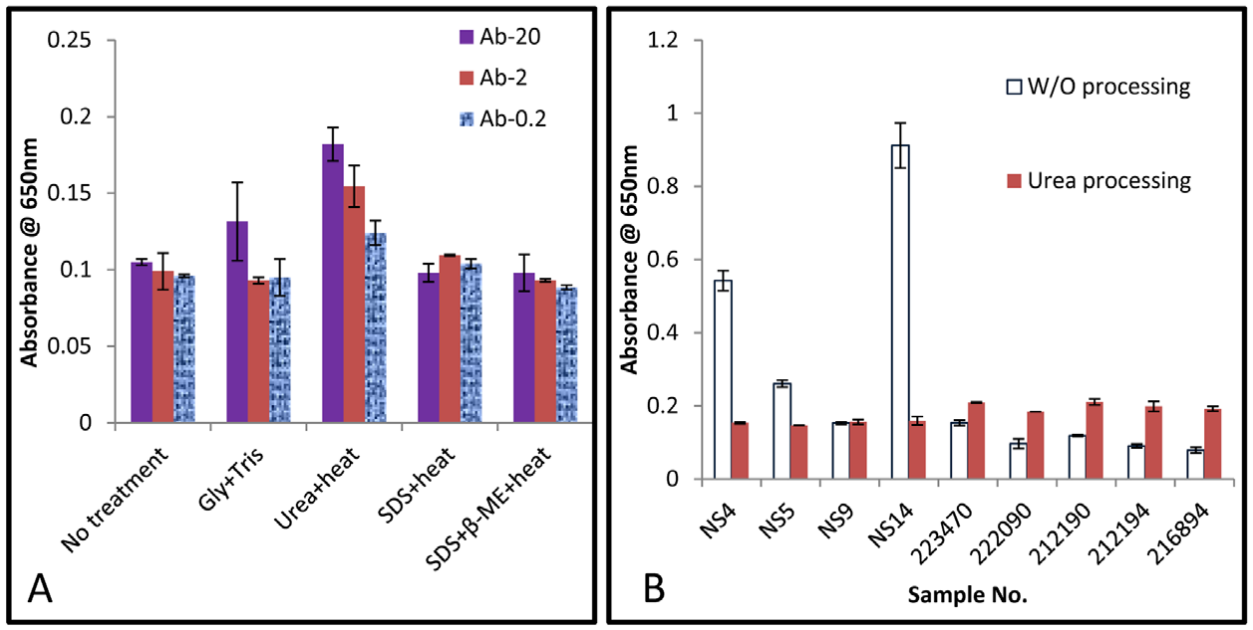
The effect of chemical treatment on the antigen detection level in the simulated and clinical samples. **A:** The effect of different chemical treatments (as mentioned along x-axis) on antigen detection level (in simulated samples). The three different antibody concentrations are mentioned at the top right hand side. Each condition (under each antibody concentration) was tested in triplicates. **B:** The antigen level detected with the clinical samples. The clear bar shows the result without (w/o) any pre-treatment while the pre-treated samples are shown via the dark bar. The first four samples were negative and rest were positive clinical samples. Each sample was tested in duplicate.

In initial studies with the clinical samples, the signal in the assay was reduced compared to the spiked samples. Based on several earlier published works, we hypothesized that this was due to the presence of immune complexes (IC) between LAM and cognate antibodies in the sera [42]–[44]. We therefore explored dissociating the IC either with acidification or with reduction or denaturation. As illustrated in Fig. 5A, in a series of test samples, among all of the conditions explored, adding urea and then heating worked best to dissociate the IC and gave the highest sensitivity. The same trend was observed in all the three Ab concentrations tested.

When applied to the clinical samples, the same effect was observed (Fig. 5B). Pre-treatment of the sample with urea enhanced the specific antigen detection signal in the positive samples (those with numerical sample number). At the same time, it reduced the background for the negative samples (those with alpha-numerical sample number).

**Figure 6:**
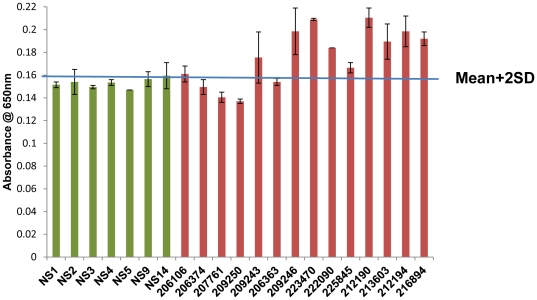
Analysis of clinical samples. The clinical samples were analysed in the microtitre plate. The line in the middle showed the cut-off point that was chosen to be mean+2 SD (standard deviation). The green bars (with alphanumeric sample number) showed the negative samples, all of which lied below the line. The red bars (with numerical sample number) showed the positive samples, nine of which were above the line.

We chose to work with the stored serum samples collected from TB patients as the designed assay showed more sensitivity in the serum matrix. Each sample was tested in duplicates. Based on the mean absorbance value of the negative samples the cut-off point was chosen as mean+2 SD (standard deviation). All the negative samples were found to be negative as they all were below the cut-off point (green bars in Fig. 6). This established 100% specificity of the assay. Of the cavitary TB samples 9 out of 14 positive samples showed an absorbance value above the cut-off point (the red bars in Fig. 6), leading to a sensitivity of 64%.

## Discussion

In this study, the LAM antigen of *M. tuberculosis* was the target for the development of an immunoswab assay to diagnose TB. *M. tuberculosis* produces copious quantities of LAM which has been shown to be secreted *in vitro* and *in vivo* LAM [45], and therefore detection of LAM can be efficiently done during the early stage of the disease. LAM has been shown to be present in not only sputum but also in blood, urine, pleural fluid and cerebrospinal fluid of *M. tuberculosis* infected patients [19]–[24]. The occurrence of LAM in body fluids other than sputum especially facilitates the detection of extra-pulmonary TB. Also, compared to sputum collection, it is relatively easy to collect blood or urine samples. The immunoswab assay reported here is capable of detecting LAM spiked in both serum and urine, indicating that the assay will be useful in detecting pulmonary, extra-pulmonary and disseminated TB.

In a study by Arias-Bouda *et. al* involving detection of LAM in sputum samples, a polyclonal antibody was employed as the detection antibody and it was found that the sensitivity and specificity of the assay were 94% and 100%, respectively [20]. In our study, an anti-LAM MAb was used to develop a bsMAb, which was then employed as the detection antibody in the assay. In comparison with polyclonal antibodies, an advantage of bsMAbs is that they can be produced with less batch-to-batch reproducibility [46]–[48], which is beneficial to the long-term robustness of an assay. In addition, as mentioned earlier (section 3.6), both MAb- and PAb- based assays require a secondary antibody–enzyme conjugate, which is typically produced by chemical conjugation, a process that is also not uniformly reproducible [49]. Moreover, because the bsMAb is already tagged with HRPO, extra steps involving secondary antibody addition and subsequent washes are avoided. This contributes to an overall reduction of time required for the immunoswab assay. Arias-Bouda *et al.* also reported a correlation between the amount of LAM and the corresponding bacterial load [20]. According to that correlation, our assay should be able to detect 5×10^3^ bacilli/ml as the detection limit for native LAM using the bsMAb was found to be 0.5 ng/ml in serum.

One LAM-ELISA kit – the Clearview TB ELISA – is commercially available although the assay has restricted sensitivity [50]–[52]. Recently, a lateral flow POC assay (Alere Determine TB) has been released and shown to be effective, especially when combined with smear-sputum microscopy [53]. Both assays usually employ urine samples and performed well in patients with advanced immune suppression, which are particularly difficult to diagnose [52]–[53]. We anticipate that should the immunoswab assay format described here prove inadequate, the bsMAb reported here may be of use in the development of lateral flow assays analogous to Alere Determine TB.

The specificity was checked with different synthetic carbohydrate antigens, as well as native carbohydrate antigens. The assay was found to be specific towards the target antigen. However, cross reactivity was detected with non-tuberculous mycobacterial LAMs.

Out of all the serum processing conditions tested, the urea and heat treatment was best to dissociate putative Ab–Ag immune complexes. This approach was superior to the use of either SDS or *β*-mercaptoethanol, which are, respectively, potent protein denaturing or reducing agents. The result can be explained by the presence of excess processing reagent during the immunoassay. For example, the presence of SDS in the processed sample could reduce signal by inhibiting the Ab–Ag attachment in the subsequent immunoassay. In contrast, the presence of excess urea was not expected to be problematic as it mainly denatures proteins at temperatures above 60°C [54]. Therefore, because we conducted the immunoassay at RT the presence of urea little interference is likely. With regard to glycine and tris treatment of samples, a report in literature that showed that this treatment can dissociate Ab–Ag complexes but that one hour of incubation is required [55]. In the interest of developing a rapid method for facilitating high throughput sample testing, we processed the samples only for 10 min, which was probably insufficient to fully dissociate immune complexes with these reagents.

The specificity (100%) of the assay was further established in the analysis with the clinical samples. The sensitivity (64%) of the assay with the clinical samples was not high. A possible explanation is that the 14 TB serum samples selected from a group of 30 cavitary TB individuals that showed the highest anti-LAM titer [34], not the presence of the antigen, which is the basis of the assay described here.

To design an assay having visually detectable end point, we transformed the assay from an ELISA plate to a swab format. The transformation reduced the sensitivity. The corresponding antigen detection limit in buffer was 0.234 ng/ml in an ELISA plate (section 3.5) and 5 ng/ml in swab (section 3.6) when performed in MAb based format. However, as noted earlier (section 3.6), the MAb based format was found to be less sensitive in the swab format than the bsMAb format. It should be noted that a limitation of the assay, or any assay relying on visual end point detection, is inter observer variability, which, in turn, influences the sensitivity. To minimize the inter observer variability, the result of the assay should always be compared with a suitable negative control; however, this issue can never be completely overcome.

The assay reported in this study is simple, easy to perform and results can be obtained within 2–3 hours of sample collection. Although the assay demands a couple of washing (fill and aspirate) and incubation steps, we believe that can be easily done by the personnel with minimal technical training. Furthermore, the cost of the reagents required to perform the assay is less than 1.00 CAD per swab (cost analysis not shown), making it inexpensive. Another advantage of this assay is that the assay endpoint can be read visually without using any sophisticated instrumentation. This type of easy, low cost and fast access to results can encourage people to start the diagnostic process [56]. It is believed that a rapid and less sophisticated detection test, even one having poor sensitivity like the SSM, could avert 15% of the annual deaths due to TB [56]. We note that the assay described in this study has all the characteristics of an ideal diagnostic test – affordable, sensitive, specific, user-friendly, rapid and robust, equipment-free and delivered to those in need (ASSURED) [57].

One of the potential limitations of the assay evaluation was that we used only 21 clinical samples and they were kept frozen for a significant time. This might have caused the wide confidence interval along with the reduced sensitivity. The use of a larger number of samples would have allowed us to evaluate the assay better. In future, we want to extend the evaluation of our assay with a greater number of freshly collected serum samples from TB and HIV co-infected patients

## References

[1] World Health Organisation website.. http://www.who.int/tb/publications/global_report/2009/pdf/report_without_annexes.pdf.

[2] Inderlied CB, Kemper A, Bermúdez LE (1993). The *Mycobacterium avium* complex.. Clin Microbiol Rev.

[3] Stop TB website.. http://www.stoptb.org/assets/documents/global/plan/SP%20Stop%20TB%20Dia%20WG%20-FINAL-Dec2005.pdf.

[4] Madariaga MG, Jalali Z, Swindells S (2007). Clinical utility of interferon gamma assay in the diagnosis of tuberculosis.. J Am Board Fam Med.

[5] Lalvani A, Millington KA (2008). Screening for tuberculosis infection prior to initiation of anti-TNF therapy.. Autoimmun Rev.

[6] Campos M, Quartin A, Mendes E, Abreu A, Guerevich S (2008). Feasibility of shortening respiratory isolation with a single sputum nucleic acid amplification test.. Am J Resp Crit Care Med.

[7] Theron G, Pooran A, Peter J, van Zyl-Smit R, Mishra HK (2011). Do adjunct TB tests, when combined with Xpert MTB/RIF, improve accuracy and the cost of diagnosis in a resource-poor setting?. Eur Respir J.

[8] Pai M, Kalantri S, Dheda K (2006). New tools and emerging technologies for the diagnosis of tuberculosis: part II. Active tuberculosis and drug resistance.. Expert Rev Mol Diagn.

[9] Dorman SE (2010). New diagnostic tests for tuberculosis: bench, bedside, and beyond.. Clin Infect Dis.

[10] Luetkemeyer AF (2010). Current issues in the diagnosis and management of tuberculosis and HIV coinfection in the United States.. Top HIV Med.

[11] Chaudhary M, Gupta S, Khare S, Lal S (2010). Diagnosis of tuberculosis in an era of HIV pandemic: a review of current status and future prospects.. Indian J Med Microbiol.

[12] Gennaro ML (2000). Immunologic diagnosis of tuberculosis.. Clin Infect Dis.

[13] Sarkar S, Suresh MR (2011). An overview of tuberculosis chemotherapy - a literature review.. J Pharm Pharm Sci.

[14] Abebe F, Holm-Hansen C, Wiker HG, Bjune G (2007). Progress in serodiagnosis of *Mycobacterium tuberculosis* infection.. Scand J Immunol.

[15] Strohmeier GR, Fenton MJ (1999). Roles of lipoarabinomannan in the pathogenesis of tuberculosis.. Microbes and Infect.

[16] Chatterjee D, Khoo KH (1998). Mycobacterial lipoarabinomannan: an extraordinary lipoheteroglycan with profound physiological effects.. Glycobiology.

[17] Russell D (1998). Release and trafficking of lipid components from mycobacterial phagosomes in infected macrophages..

[18] Briken V, Porcelli SA, Besra GS, Kremer L (2004). Mycobacterial lipoarabinomannan and related lipoglycans: from biogenesis to modulation of the immune response.. Molec Microbiol.

[19] Sada E, Aguilar D, Torres M, Herrera T (1992). Detection of lipoarabinomannan as a diagnostic test for tuberculosis.. J Clin Microbiol.

[20] Arias-Bouda LMP, Nguyen L, Ho L, Kuijper S, Jansen H (2000). Development of antigen detection assay for diagnosis of tuberculosis using sputum samples.. J Clin Microbiol.

[21] Boehme C, Molokova E, Minja F, Geis S, Loscher T (2005). Detection of mycobacterial LAM with an antigen capture ELISA in unprocessed urine of Tanzanian patients with suspected tuberculosis.. Trans R Soc Trop Med Hyg.

[22] Patel VB, Singh R, Connolly C, Kasprowicz V, Zumla A (2010). Comparison of a clinical prediction rule and a LAM antigen-detection assay for the rapid diagnosis of TBM in a high HIV prevalencesetting.. PLoS One.

[23] Dheda K, Van-Zyl Smit RN, Sechi LA, Badri M, Meldau R (2009). Clinical diagnostic utility of IP-10 and LAM antigen levels for the diagnosis of tuberculous pleural effusions in a high burden setting.. PLoS One.

[24] Patel VB, Bhigjee AI, Paruk HF, Singh R, Meldau R (2009). Utility of a novel lipoarabinomannan assay for the diagnosis of tuberculous meningitis in a resource –poor high-HIV prevalence setting.. Cerebrospinal Fluid Res.

[25] Mahadevan S (1997). Clinical utility of serodiagnosis of tuberculosis.. Indian J Pediatr.

[26] Bhatnagar P K, Das D, Suresh MR (2008). Sequential affinity purification of peroxidase tagged bispecific anti-SARS CoV antibodies on phenylboronic acid agarose.. J Chromatogr B Analyt Technol Biomed Life Sci.

[27] Gadikota RR, Callam CS, Appelmelk BJ, Lowary TL (2003). Synthesis of oligosaccharide fragments of mannosylated lipoarabinomannan appropriately functionalized for neoglycoconjugate preparation.. J Carbohydr Chem.

[28] Murase T, Zheng RB, Joe M, Bai Y, Marcus SL (2009). Structural insights into antibody recognition of mycobacterial polysaccharides.. J Mol Biol.

[29] Kaur D, Lowary TL, Vissa VD, Crick DC, Brennan PJ (2002). Characterization of the epitope of anti-lipoarabinomannan antibodies as the terminal hexaarabinofuranosyl motif of mycobacterial arabinans.. Microbiology.

[30] Hunter SW, Gaylord H, Brrennan PJ (1986). Structure and antigenicity of the phosphorylated lipopolysaccharide antigens from the leprosy and tuberle bacilli.. J Biol Chem.

[31] Rademacher C, Shoemaker GK, Kim H, Zheng RB, Taha H (2007). Ligand specificity of CS-35, a monoclonal antibody that recognizes mycobacterial lipoarabinomannan: a model system for oligofuranoside-protein recognition.. J Am Chem Soc.

[32] Tong M, Jacobi CE, van de Rijke FM, Kuijper S, van de Werken S (2005). A multiplexed and miniaturized serological tuberculosis assay identifies antigens that discriminate maximally between TB and non-Tb sera.. J Immunol Methods.

[33] The Tuberculosis Trial Consortium (2002). Rifapentine and isoniazid once a week versus rifampicin and isoniazid twice a week for treatment of drug-susceptible pulmonary tuberculosis in HIV-negative patients: a randomised clinical trial.. Lancet.

[34] Spencer JS, Kim HJ, Wheat WH, Chatterjee D, Balagon MV (2011). Analysis of antibody responses to Mycobacterium leprae phenolic glycolipid I, lipoarabinomannan, and recombinant proteins to define disease subtype-specific antigenic profiles in leprosy.. Clin Vaccine Immunol.

[35] Shahhosseini S, Das D, Qiu X, Feldmann H, Jones SM (2007). Production and characterization of monoclonal antibodies against different epitopes of Ebola virus antigens.. J Virol Methods.

[36] Shahhosseini S, Guttikonda S, Bhatnagar P, Suresh MR (2006). Production and characterization of monoclonal antibodies against shope fibroma virus superoxide dismutase and glutathione-s-transferase.. J Pharm Pharmaceut Sci.

[37] Das D, Suresh MR (2005). Producing bispecific and bifunctional antibodies.. Methods Mol Med.

[38] Tang XL, Peppler MS, Irvin RT, Suresh MR (2004). Use of bispecific antibodies in molecular velcro assays whose specificity approaches the theoretical limit of immunodetection for *Bordetella pertusis*.. Clin Diag Lab Immunol.

[39] Kreutz FT, Xu D, Suresh MR (1998). A new method to generate quadromas by electrofusion and FACS sorting.. Hybridoma.

[40] Suresh MR, Cuello AC, Milstein C (1986). Bispecific monoclonal antibodies from hybrid hybridomas.. Methods Enzymol.

[41] Guttikonda S, Tang XL, Yang BM, Armstrong GD, Suresh MR. Monospecific and bispecific antibodies against E. coli O157 for diagnostics.. J Immunol Methods.

[42] Miles SA, Balden E, Magpantay L, Wei L, Leiblein A (1993). Rapid serologic testing with immune-complex dissociated HIV p24 antigen for early detection of HIV infection in neonates. Southern California Pediatric AIDS Consortium.. N Engl J Med.

[43] Krijger FW, van Lieshout L, Deelder AM (1994). A simple technique to pretreat urine and serum samples for quantitation of schistosome circulating anodic and cathodic antigen.. Acta Trop.

[44] Koraka P, Burghoorn-Maas CP, Falconar A, Setiati TE, Djamiatun K (2003). Detection of immune-complex-dissociated nonstructural-1 antigen in patients with acute dengue virus infections.. J Clin Microbiol.

[45] Józefowski S, Sobota A, Kwiatkowska K (2008). How *Mycobacterium tuberculosis* subverts host immune responses.. BioEssays.

[46] Lipman NS, Jackson LR, Trudel LJ, W-Garcia F (2005). Monoclonal versus polyclonal antibodies: distinguishing characteristics, applications and information resources.. Inst Lab Anim Res J.

[47] Suresh MR, Cuello AC, Milstein C (1986). Advantages of bispecific hybridomas in one-step immunocytochemistry and immunoassays.. Proc Natl Acad Sci U S A.

[48] Ritter MA (2000). Polyclonal and monoclonal antibodies.. Methods Mol Med.

[49] Cao Y, Suresh MR (1998). Bispecific antibodies as novel bioconjugates.. Bioconjug Chem.

[50] Reither K, Saathoff E, Jung J, Minja LT, Kroidl I (2009). Low sensitivity of a urine LAM-ELISA in the diagnosis of pulmonary tuberculosis.. BMC Infect Dis.

[51] Dheda K, Davids V, Lenders L, Roberts T, Meldau R (2010). Clinical utility of a commercial LAM-ELISA assay for TB diagnosis in HIV-infected patients using urine and sputum samples.. PLoS One.

[52] Peter J, Green C, Hoelscher M, Mwaba P, Zumla A (2010). Urine for the diagnosis of tuberculosis: current approaches, clinical applicability,and new developments.. Curr Opin Pulm Med.

[53] Lawn SD, Kerkhoff AD, Vogt M, Wood R (2011). Diagnostic accuracy of a low-cost, urine antigen, point-of-care screening assay for HIV-associated pulmonary tuberculosis before antiretroviral therapy: a descriptive study.. Lancet Infect Dis.

[54] Bennion BJ, Daggett V (2003). The molecular basis for the chemical denaturation of proteins by urea.. Proc Natl Acad Sci U S A.

[55] Vasudevachari MB, Salzman NP, Woll DR, Mast C, Uffelman KW (1993). Clinical utility of an enhanced human immunodeficiency virus type 1 p24 antigen capture assay.. J Clin Immunol.

[56] Keeler E, Perkins MD, Small P, Hanson C, Reed S (2006). Reducing the global burden of tuberculosis: the contribution of improved diagnostics.. Nature.

[57] Mabey D, Peeling RW, Ustianowski A, Perkins MD (2004). Diagnostics for the developing world.. Nat Rev Microbiol.

